# Sequential multi-reconstructive approach to a massive scalp defect: a case report

**DOI:** 10.1093/jscr/rjaf778

**Published:** 2025-10-08

**Authors:** Luz Elena Rueda Gallardo, Juan Pablo Zaraza Duarte, Fabien Catherine Luna Aguilar, Frank Andrés Álvarez Vásquez

**Affiliations:** Universidad Nacional de Colombia, Bogotá DC, Colombia; Universidad del Rosario, Bogotá DC, Colombia; Plastic and Reconstructive Surgery Resident at Universidad Nacional, Bogotá DC, Colombia; Universidad del Rosario, Bogotá DC, Colombia

**Keywords:** scalp, trephining, dermal matrix

## Abstract

Reconstruction of scalp defects continues to be a surgical challenge due to its anatomical and functional characteristics. The ideal reconstructive technique between local flaps, tissue expanders, dermal matrices, the use of negative pressure system and free flaps should provide durable and aesthetically acceptable coverage based on the etiology, defect size, local tissue availability, and patient comorbidities; with the aim of preserving hairlines, coverage with hair-bearing skin and preventing antiesthetic scars and areas of alopecia. We present a case of scalp avulsion affecting 90% of the total area, in which multiple surgical strategies were used to achieve the stated objective.

## Introduction

The scalp acts as a protective barrier for cranial structures, preventing desiccation and infection and is important for aesthetic appearance. Reconstruction of this tissue after avulsive trauma should ensure a stable coverage that resembles the characteristics of the original tissue, preserving the hairline and minimizing unsightly scars and areas of alopecia. Scalp reconstruction is complex and must be tailored to the etiology of the trauma, the size of the defect, the availability of local tissue, comorbidities of the patient, and the morbidity of the donor area.

In cases of large defects, free flaps are usually the first option [1–3]. However, it is important to evaluate additional reconstructive strategies that can lead to better aesthetic and functional outcomes, associated with less morbidity. We present a clinical case of scalp avulsion secondary to trauma by machine pulling and the surgical process by stages to achieve its reconstruction.

## Case report

**Figure 1:**
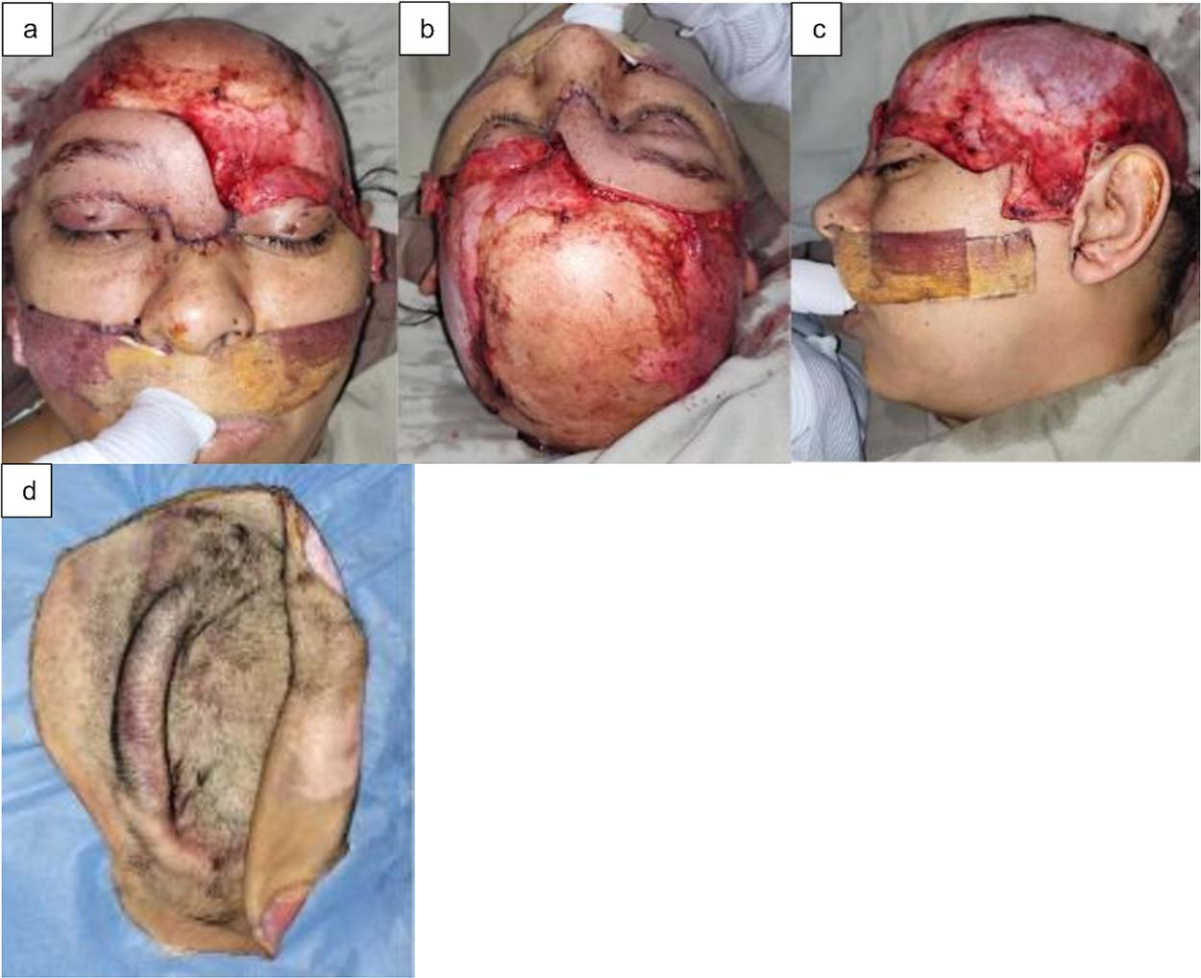
(a) Frontal view, (b) superior view, (c) left lateral view of the scalp avulsion, and (d) avulsed scalp.

A 45-year-old female with a 90% scalp avulsion and a pedicled avulsion flap in the temporal region including the orbital portion of the right upper eyelid and the ipsilateral eyebrow was admitted in hypovolemic shock. The scalp avulsion flap was found to be intact and cold preservation was initiated (Fig. 1).

**Figure 2:**
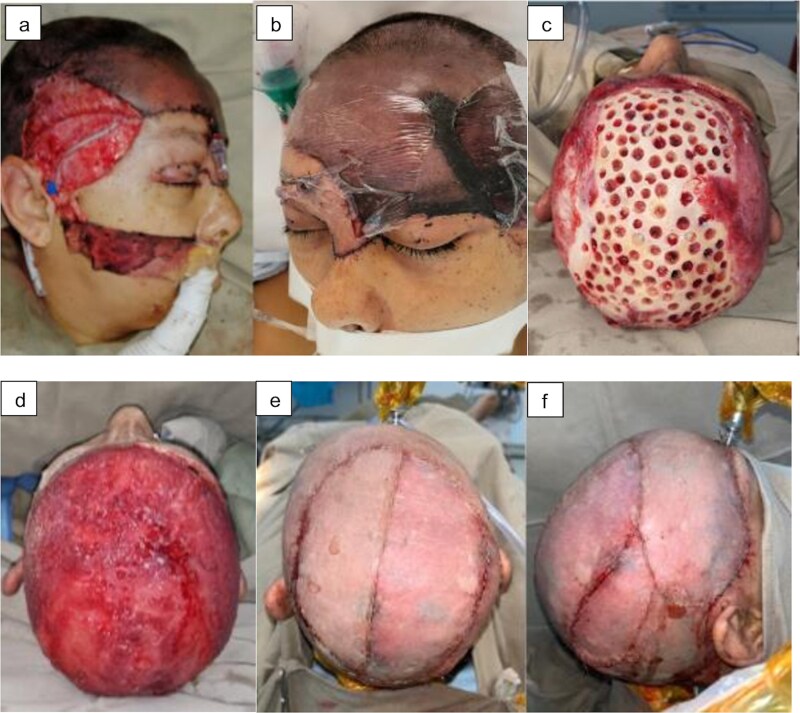
Sequential multi-reconstructive approach: (a) microsurgical reimplantation, (b) total necrosis of the flap, (c) trepanations, (d) coverage with dermal matrix, (e) superior view of the partial skin grafts, and (f) right lateral view.

Given the ischemia time of <4 hours, reimplantation was performed with two arterial anastomoses (superficial temporal arteries) and two venous anastomoses (superficial temporal veins, requiring saphenous graft for the venous bridge). However, on the second day, there was thrombosis of all anastomoses with consequent total necrosis of the flap, debridement was performed, facing again a complex craniofacial coverage defect and it was decided to performer a staged reconstruction (Fig. 2a and b).

1. *First stage—trephining and subatmospheric pressure system:* Trepanations in the entire external table, up to the diploe, associated with negative pressure to favor the formation of granulation tissue, avoiding its desiccation (Fig. 2c).
2. *Second stage—full thickness graft and periodic changes of the subatmospheric pressure system*: Once adequate granulation tissue was obtained, a full thickness inguinal graft was used to cover the orbital portion of the left upper eyelid and the subunit of the left eyebrow. Seven weekly lavages and changes of the negative pressure system were necessary to complete the total granulation of the exposed skull.
3. *Third stage—dermal matrix*: Matriderm-2mm® was placed and capitoneated with negative pressure system favoring its integration (Fig. 2d).
4. *Fourth stage—partial skin grafts*: One week later partial thigh skin grafts were used to cover the total defect using the Mayfield support that allows a 360° cranial approach and again the negative pressure system was used to capitonize, ensuring the adequate integration of the grafts (Fig. 2e and f).

**Figure 3:**
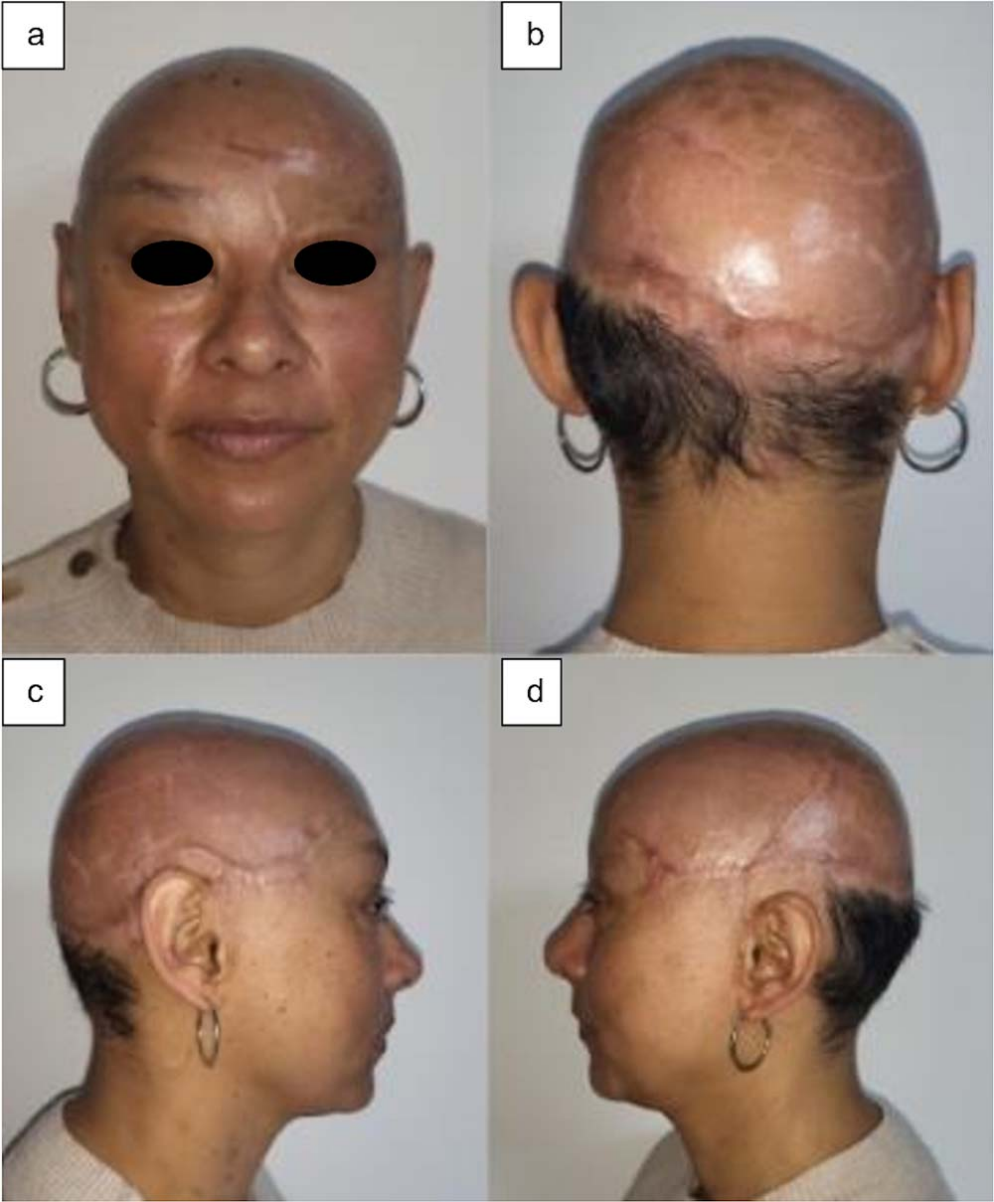
Postoperative outcomes at the fifth month: (a) anterior view, (b) posterior view, (c) right lateral view, and (d) left lateral view.

At 5 months postoperatively, the patient presented a favorable esthetic result with and adequate reconstruction of the subunit; the tissue was stable, allowing the use of a capillary prosthesis. At 12 months postoperatively, the result indicated an excellent integration of the grafts and dermal matrices, as well as a satisfactory recovery of the craniofacial reconstruction (Fig. 3).

## Discussion

Complete scalp avulsion is a rare, potentially life-threating injury that usually occurs when the long hair is pulled by any mechanical force, being more frequent in women. Data on its epidemiology are limited [4].

Microsurgical reimplantation is the standard treatment because it maintains the scalp’s own skin and achieves the best aesthetic and functional results, indicated only if the avulsed flap has a warm ischemia time of <30 h, has both recipient vessels and flap vessels intact and has an adequate chain of conservation. The first successful reimplantation was reported by Miller *et al*. in 1976 [2, 5]. Even orthotopic reimplantation in a second surgical time has been described with only three case reports in the medical literature [6]. Among the most outstanding microsurgical options are the greater omentum flap and the latissimus dorsi flap. The first offers a rich vascular network and is versatile to cover irregular areas; however, it is a complex procedure that requires a second abdominal incision and generates high morbidity at the donor site. The second provides a significant amount of tissue, suitable for extensive and deep defects, but is also associated with high morbidity [2, 3, 7].

Partial thickness grafts are less invasive and also has less morbidity at the donor site to ensure their adequate integration, it is crucial to have a nutrient-rich and well vascularized recipient bed, to achieve this goal in an uncovered calvaria, the following strategies have been described [1, 2]:

- Periosteal flaps, although effective, are not available in all cases [8].
- Trepanation of the external cortex of the cranial vault exposes the diploe and promotes the formation of granulation tissue that emerges through these holes to cover the calvarium. In 1904, Mellish reported on the first method of trepanation that was documented in the year 1777 [9].
- Negative pressure therapy allows continuous and automatic cleaning of the area, provides isolation from the external environment, reduces dead space and edema, and promotes cell proliferation, angiogenesis, and wound contraction, with less need for surgical debridement; contraindicated in highly contaminated wounds, active bleeding, and osteomyelitis [1].

These grafts may present a high risk of pathological scarring, contour deformities, hypopigmentation, and ulcerations, with frequently unsatisfactory aesthetic results [1, 2] To improve these results, the use of dermal matrices has been shown to be beneficial, promoting the formation of a well vascularized neodermis that contributes to decrease the formation of visible and prominent scars, facilitate more uniform healing by reducing contractures that can affect appearance and functionality, promote a more flexible and adaptable skin, resulting in a more natural appearance and functional recovery [8, 10].

Based on the aforementioned precepts, it was decided to reconstruct the massive scalp defect presented in sequential steps (four stages after the failed microsurgical reimplantation) to achieve the most adequate aesthetic and functional result.

## Conclusion

Staged reconstruction, using trephination, negative pressure system, dermal matrices, and skin grafts, is an effective strategy to reconstruct extensive scalp defects. Continuous evaluation and proper management of each stage is crucial to ensure a successful reconstruction and minimize complications. A multidisciplinary approach, including plastic surgery, intensive care, and rehabilitation, is essential to optimize results.
